# Processed Food and Atopic Dermatitis: A Pooled Analysis of Three Cross-Sectional Studies in Chinese Adults

**DOI:** 10.3389/fnut.2021.754663

**Published:** 2021-12-06

**Authors:** Yajia Li, Juan Su, Dan Luo, Yanying Duan, Zhijun Huang, Meian He, Juan Tao, Shuiyuan Xiao, Yi Xiao, Xiang Chen, Minxue Shen

**Affiliations:** ^1^Department of Dermatology, Hunan Engineering Research Center of Skin Health and Disease, Hunan Key Laboratory of Skin Cancer and Psoriasis, Xiangya Hospital, Central South University, Changsha, China; ^2^Department of Social Medicine and Health Management, Xiangya School of Public Health, Central South University, Changsha, China; ^3^Department of Occupational and Environmental Health, Xiangya School of Public Health, Central South University, Changsha, China; ^4^Center of Clinical Pharmacology, The Third Xiangya Hospital, Central South University, Changsha, China; ^5^Department of Occupational and Environmental Health, State Key Laboratory of Environmental Health for Incubating, School of Public Health, Tongji Medical College, Huazhong University of Science and Technology, Wuhan, China; ^6^Department of Dermatology, Tongji Medical College, Union Hospital, Huazhong University of Science and Technology, Wuhan, China

**Keywords:** atopic dermatitis, processed meat, pickles, sodium, adult

## Abstract

**Objective:** The effect of processed foods on atopic dermatitis (AD) in adults is unclear. This study was to evaluate the association between processed foods and AD in the Chinese adult population.

**Design:** This study included three population-based cross-sectional studies using cluster sampling by villages, institutions, or factories. Participants underwent dermatological examinations by certificated dermatologists and a food frequency questionnaire survey. A spot urine sample was collected to estimate the daily sodium intake. Adjusted odds ratios (aORs) and 95% confidence intervals (CIs) were presented as the effect size.

**Setting:** Shiyan city of Hubei province, and Huayuan, Shimen, Hengyang, Zhuzhou, and Changsha of Hunan province.

**Participants:** Automobile manufacture workers from Shiyan of Hubei province, and rural residents and civil servants from Hunan.

**Results:** A total of 15,062 participants, including 3,781 rural residents, 5,111 civil servants, and 6,170 workers, completed all evaluations. Compared to those hardly consumed pickles, consumption of pickles 1–3 times per week was significantly associated with AD (aOR: 1.35; 95% CI: 1.06–1.70). The intake of processed meats 1–3 times per month (aOR: 1.29; 95% CI: 1.05–1.58) and 1–3 times per week (aOR: 1.44; 95% CI: 1.11–1.87) were associated with AD dose-dependently when compared with those who rarely ate processed meats. Compared with non-consumers, the consumption of any processed foods 1–3 times per week (aOR: 1.39; 95% CI: 1.08–1.80) and ≥4 times per week (aOR: 1.41; 95% CI: 1.05–1.89) showed increased risks of AD. A positive association of estimated sodium intake with AD was also observed.

**Conclusion:** Intake of processed foods is associated with AD in Chinese adults.

## Introduction

Atopic dermatitis (AD), also known as atopic eczema, is a frequently occurring, complex, chronic inflammatory skin disease, which is characterized by recurrent pruritus, xerosis, and eczematous lesions. It is originally regarded as a childhood disease with defects of the barrier function of the epidermis, Th1/2 immune response dysregulation, and the elevated serum IgE level (1, 2). The prevalence of AD ranges from 2 to 17.6% in adults worldwide with significant heterogeneities across populations, regions, and research methods. With the changes in people's lifestyles and environment, an increasing trend in the incidence and prevalence of AD was identified (3, 4). While there are few reports on the epidemiology of adult AD, it is not uncommon to identify adult-onset patients clinically.

Risk factors for adult AD are complex. Many hypotheses have been proposed from the perspectives of hygiene, diversity of intestinal flora, exposure to endotoxins through farm animals to pollution, climate, and diet (5). Recently, more and more studies have concerned the associations among dietary structure, nutrients, and AD. Studies have shown that adherence to a Mediterranean diet and consumption of fermented milk products might be protective factors for eczema, (6, 7) while there is also evidence that the preference of a “Western” diet which includes rich intake of refined grains, red meat, cured food, and saturated or unsaturated fatty acids may be related to an increased risk in dermatitis and cutaneous inflammation (8). However, the role of dietary patterns and food preferences in the Chinese population remains to be clarified, because the huge differences between Chinese and Western diets make the conclusions of previous studies impossible to be well generalized. Processed foods (including processed meats, pickles, etc.) are important components in Western dietary patterns and also constitute a part of the traditional Chinese family diet, especially in rural areas (9). The preparation of processed food is packing moist vegetables tightly in a jar or air drying meat/fish/vegetables with some spices for a few weeks to months, while salt is ubiquitously used in the procedure of processing and preserving (10). The global consumption of processed meat and salt is higher than the global optimal level, while sodium consumption has exceeded the optimal in almost every region of the world (11–13). High-sodium diets are one of the leading risk factors of death and non-infectious diseases (14, 15). However, there is lack of the research on the association of processed foods and salt intake with AD.

The current study aimed to evaluate the associations of the intake of processed foods and salt with the prevalence of adult AD based on cross-sectional data obtained from three population-based studies in China.

## Methods

### Study Population

This is a pooled analysis of three cross-sectional studies that were conducted using comparable methodologies during 2016 and 2019. The study population comprised of:

(1) Automobile manufacture workers from Shiyan city of Hubei province who are participants of the Dongfeng-Tongji Cohort Study (16): the cohort was established in 2008 and initially recruited retired workers, and the cohort began to recruit in-service workers since 2016; in the current analysis, we used the baseline data collected from the in-service workers.
(2) Rural residents from Huayuan, Shimen, Hengyang, and Zhuzhou cities or counties of Hunan province who are participants of the Hunan Rural Resident Chronic Disease Study (17, 18): the study was established in 2016 and recruited residents in rural regions through cluster sampling by the village.
(3) Civil servants from Changsha city of Hunan province who are participants of the Hunan Government Employee Health Study (19, 20): the study was established in 2017 and recruited civil servants in urban regions through cluster sampling by institutions.

The review committee of Xiangya Hospital and Xiangya School of Public Health of Central South University (approval number# XYGW-2016-10) and Tongji Medical College of Huazhong University of Science and Technology has approved the studies (approval number# 2016-IEC-S128). Written informed consent was obtained from all participants.

### Exposure Assessment

The intakes of processed meats and pickles were inquired through the semi-quantitative food frequency questionnaire (FFQ). The FFQ questionnaire survey adopted a face-to-face inquiry method, and the inquiry was conducted and recorded by trained investigators, and it took 10–15 min per participant. The items of red meat, poultry, fish, milk, egg, vegetable, fruit, processed meat, and pickles were included in the questionnaire. The cohort was carried out in similar periods in different regions, and the same FFQ survey tool was used in the cohort. Besides, in consideration of the difference between western and Chinese diets, as well as according to the following definition: meat that were cooked through salting, smoking, or other processes for flavor or preservation improvement, (21) processed meat in this study included: smoked/cured/roast meat or beef, sausage, luncheon meat, etc., which are the common traditional processed styles in the Chinese diet. Pickles included pickled mustard tubers, salted vegetables, and sauce vegetables, etc.

There are four categories included in the frequency of intake: hardly, 1–3 times/mon, 1–3 times/week, and ≥4 times/week. The daily sodium intake from processed foods was estimated according to previous studies that determined the salt contents in different processed foods in Hunan and Hubei regions.

Spot urine sample was also collected from each individual to evaluate the intake of sodium. The method proposed by Tanaka et al. was used to estimate the 24 h urinary sodium excretion (24HUNa) by using spot urine samples (22). The formulas are shown as follows:

(1) PrUCr = 14.89 × Weight + 16.14 × Height – 2.04×age – 2244.45
(2) 24HUNa=23 × 21.98 × (SUNa/SUCr × PRCr)0.392

In the formulas, PrUCr is the predicted value of 24 h urinary creatinine (mg/day), SUNa is the concentration of sodium (mmol/L) in the spot voiding urine, and SUCr is the concentration of creatinine (mmol/L) in the spot voiding urine. Weight (kg) and height were measured by research nurses. Age (year) was self-reported through the questionnaire survey.

### Outcome Assessment

AD was diagnosed by certificated dermatologists during the field health examination, according to the guideline from the American Academy of Dermatology (23). We collected information on clinical manifestation, history of diseases, and family history from all participants.

### Assessment of Covariates

Standardized methods were used in the measurement of height, weight, and blood pressure. Body mass index (BMI) was calculated as weight (kg) / squared height (m^2^). Mean arterial pressure (MAP) was calculated as (systolic pressure + 2 × diastolic pressure)/3. Age, sex, household income, educational level, smoking status, and alcohol drinking were collected through the questionnaire. The intakes of other foods including red meat, poultry, fish, egg, milk, fruit, vegetable, etc. were determined by the FFQ.

### Statistical Analysis

Mean ± standard deviation (SD) was used to present the continuous data, and analysis of variance (ANOVA) was used in the between-group difference test. Number (%) was shown in presenting the categorical data, and the chi-square test was used for comparing the between-group difference.

Generalized weighted quantile sum (gWQS) regression was used to select AD-related dietary factors, and a linear connection function was used to connect the resulting average to the weighted sum of exposure quartiles and covariates (24). The gWQS regression can not only evaluate the overall impact of food while taking into account collinearity, but it can also determine the main dietary factors that lead to the observed correlation.

Three-level logistic regression models were used for estimating the association of processed food with AD, adjusting for the fixed effects of level-1 covariates (age, sex, income, smoking, intake of other foods, BMI, and MAP) and the random effects of level-2 (villages/institutions/factories) and level-3 (studies) units. Adjusted odds ratio (aOR) and 95% confidence interval (CI) were used to present the effect size.

We used the generalized additive models with logit link function and binomial distribution to estimate the association of sodium intake and prevalence of AD. The above-mentioned confounders were adjusted in models and cubic splines were used for smoothing. For all tests, a *P* value < 0.05 was statistically significant. SAS 9.4 (SAS Institute Inc., Cary, USA) was used in performing the statistical analysis.

## Result

**Figure 1:**
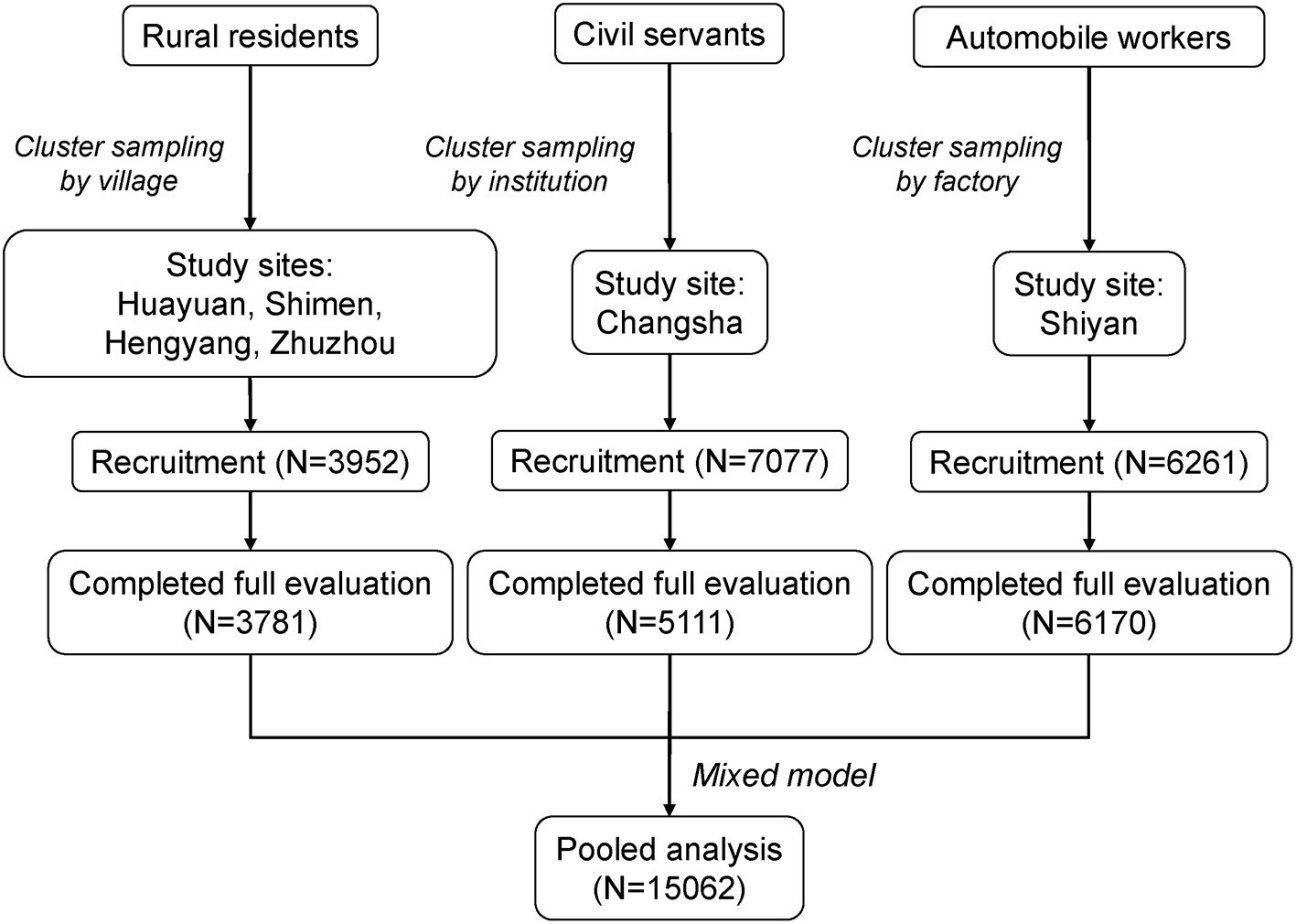
Flow chart of the inclusion of participants.

A total of 17,290 participants were recruited at baseline. Among them, 15,062 consented to participate and underwent all evaluations, including 3,781 rural residents, 6,170 workers, and 5,111 civil servants (Figure 1). The crude point prevalence of AD was 1.59, 3.14, and 6.46% in rural residents, workers, and civil servants, respectively, indicating a gradient of prevalence in accordance with socioeconomic status. The baseline characteristics were statistically different across the three populations. Annual house income and education were collected as categorical variables, while BMI and MAP were collected as continuous variables (Table 1). Rural residents had the lowest income and education levels and the highest MAP. In contrast, civil servants reported the highest income and education levels and the least proportion of current and past smoking.

**Table 1:** Characteristics of the participants in three studies in China.

**Characteristics**	**Total**	**Stratified by study**		
		**Rural residents**	**Civil servants**	**Workers**
Age (years), mean (SD)	44.5 (11.9)	52.0 (13.7)	41.3 (11.8)	42.4 (8.5)
**Sex**, ***n*** **(%)**				
Male	8,769 (58.2)	2,176 (57.6)	2,156 (42.2)	4,437 (71.9)
Female	6,293 (41.8)	1605 (42.4)	2955 (57.8)	1,733 (28.1)
**Annual house income (CNY)**, ***n*** **(%)**				
<30,000	2,633 (17.5)	2,310 (61.1)	0	323 (5.2)
30,000–49,999	3,160 (21.0)	879 (23.2)	656 (12.8)	1,625 (26.3)
50,000–99,999	4,718 (31.3)	484 (12.8)	1,313 (25.7)	2,921 (47.3)
100,000–199,999	3,013 (20.0)	89 (2.4)	1,828 (35.8)	1,096 (17.8)
≥200,000	1,538 (10.2)	19 (0.5)	1,314 (25.7)	205 (3.3)
**Education**, ***n*** **(%)**				
Primary school and below	1,330 (8.8)	1,282 (33.9)	16 (0.3)	32 (0.5)
Middle school	1,384 (9.2)	1,014 (26.8)	86 (1.7)	284 (4.6)
High school	3,880 (25.8)	978 (25.9)	297 (5.8)	2,605 (42.2)
College and above	8,468 (56.2)	507 (13.4)	4,712 (55.6)	3,249 (52.7)
**Smoking status**, ***n*** **(%)**				
Non-smoker	10,638 (70.6)	2,286 (60.5)	4,441 (86.9)	3,911 (63.4)
Current smoker	3,592 (23.8)	1140 (30.1)	565 (11.1)	1,887 (30.6)
Past smoker	832 (5.5)	355 (9.4)	105 (2.0)	372 (6.0)
**Alcohol drinking**				
Non-drinker	11,154 (74.0)	2,982 (78.9)	4,495 (87.9)	3,677 (59.6)
Current drinker	3,474 (23.0)	618 (16.3)	551 (10.8)	2,305 (37.4)
Past drinker	434 (2.8)	181 (4.8)	65 (1.3)	188 (3.0)
Body mass index (kg/m^2^), mean (SD)	23.8 (3.0)	23.9 (3.3)	23.3 (3.1)	24.1 (2.6)
Mean arterial pressure (mmHg), mean (SD)	94.2 (13.1)	100.0 (14.2)	87.2 (11.0)	96.4 (11.3)

**Figure 2:**
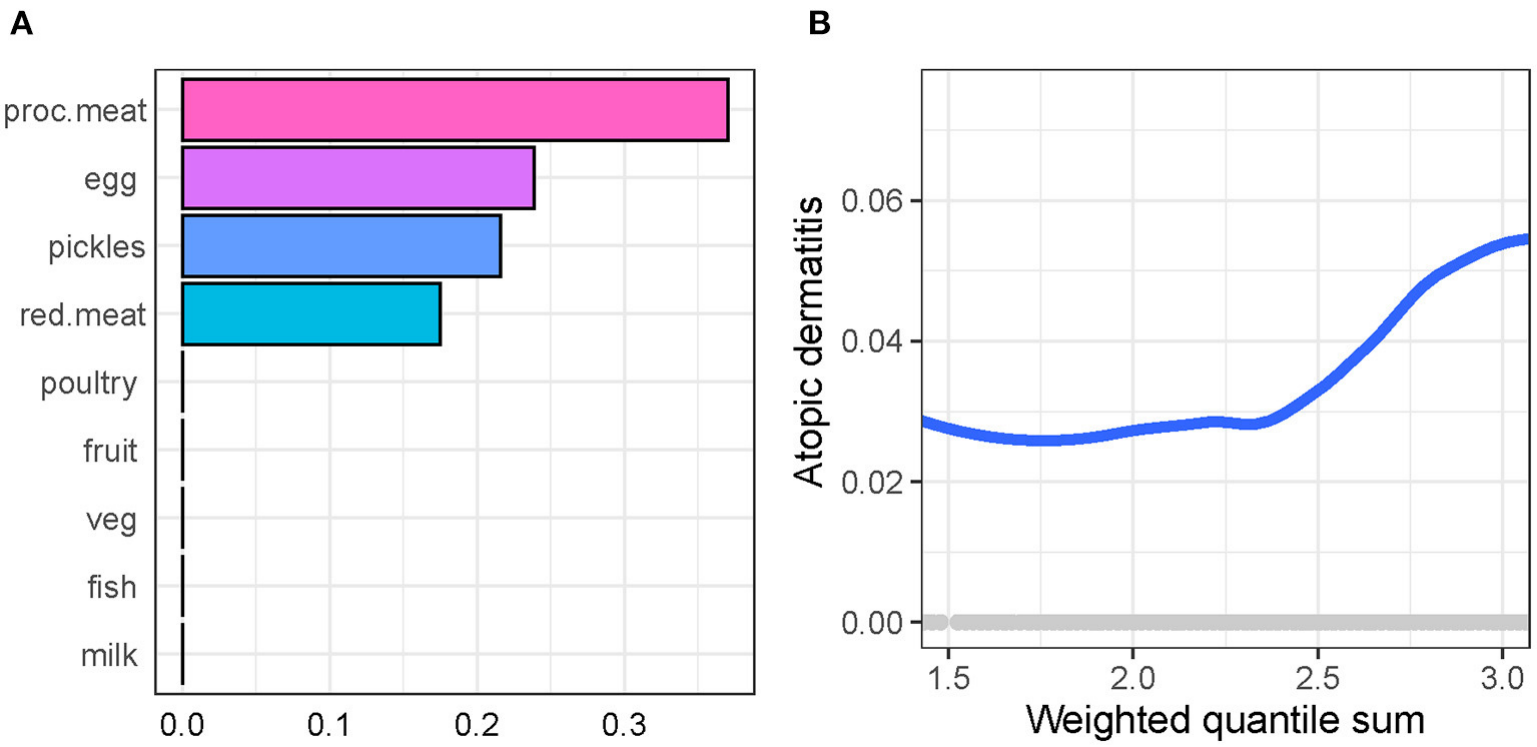
Generalized weighted quantile sum regression to select atopic dermatitis-related dietary factors in participants. **(A)** The rank of importance of food in their association with atopic dermatitis. **(B)** The association of weighted quantile sum (a synthesized score of food intake weighted by the importance of food) with atopic dermatitis.

We used gWQS regression to select AD-related dietary factors. Figure 2 shows the rank of importance of food type in their association with AD, where processed meat shows the largest effect size, followed by egg, pickles, and red meat.

Compared to non-consumers, participants who consumed pickles 1–3 times per week (aOR = 1.35, 95% CI: 1.06–1.70, *P* = 0.014) had a greater risk of AD after adjusting for covariates. Intake of processed meat 1–3 times per month (aOR = 1.29, 95% CI: 1.05–1.58, *P* = 0.014) and 1–3 times per week (aOR = 1.44, 95% CI: 1.11–1.87, *P* = 0.006) were associated with AD dose-dependently. The consumption of any processed food 1–3 times per week (aOR = 1.39, 95% CI: 1.08–1.80, *P* = 0.011) and ≥4 times per week (aOR = 1.41, 95% CI: 1.05–1.89, *P* = 0.023) also showed increased risks of AD (Table 2).

**Table 2:** Association of pickles and bacon intake frequency with atopic eczema in Chinese participants.

**Exposure**	**Frequency**	**AD (%)**	**Model 1**		**Model 2**		**Model 3**	
			aOR (95% CI)^*^	***P***	aOR (95% CI)^*^	***P***	aOR (95% CI)^*^	***P***
Pickles	Hardly	136 (3.1)	Reference		Reference		Reference	
	1–3 times/month	222 (3.9)	1.13 (0.91, 1.41)	0.269	1.12 (0.90, 1.40)	0.313	1.12 (0.90, 1.40)	0.321
	1–3 times/week	176 (4.7)	1.38 (1.09, 1.74)	0.007	1.35 (1.07, 1.71)	0.012	1.35 (1.06, 1.70)	0.014
	≥4 times/week	50 (3.9)	1.09 (0.78, 1.52)	0.611	1.09 (0.77, 1.53)	0.632	1.08 (0.77, 1.52)	0.663
Processed meat	Hardly	176 (3.2)	Reference		Reference		Reference	
	1–3 times/month	282 (4.1)	1.31 (1.07, 1.61)	0.008	1.30 (1.06, 1.59)	0.012	1.29 (1.05, 1.58)	0.014
	1–3 times/week	105 (4.8)	1.46 (1.13, 1.90)	0.004	1.45 (1.11, 1.88)	0.006	1.44 (1.11, 1.87)	0.006
	≥4 times/week	21 (3.8)	1.01 (0.63, 1.62)	0.951	1.02 (0.63, 1.64)	0.943	1.01 (0.63, 1.64)	0.954
Processed foods	Hardly	7 (1.6)	Reference		Reference		Reference	
	1–3 times/month	40 (3.2)	1.32 (0.98, 1.78)	0.068	1.32 (0.98, 1.78)	0.071	1.31 (0.97, 1.77)	0.077
	1–3 times/week	160 (4.0)	1.41 (1.10, 1.82)	0.007	1.40 (1.08, 1.80)	0.01	1.39 (1.08, 1.80)	0.011
	≥4 times/week	376 (4.0)	1.42 (1.07, 1.90)	0.016	1.42 (1.06, 1.90)	0.021	1.41 (1.05, 1.89)	0.023

* *Model 1 was adjusted for age and sex; model 2 was further adjusted for income, smoking, intake of red meat and poultry; model 3 was further adjusted for BMI and MAP*.

In the subgroup analysis by study (Table 3), the findings are generally consistent, although some of the correlations are not significant owing to smaller sample sizes. The consumption of pickles ≥4 times per week in rural residents showed an increased risk of AD (aOR = 2.95, 95% CI: 1.36–6.41, *P* = 0.006). The intake of processed meat 1–3 times per week showed a positive association with AD in workers (aOR = 1.71, 95% CI: 1.06–2.74, *P* = 0.027).

**Table 3:** Association of pickles and bacon intake frequency with AD by study.

**Exposure**	**Frequency**	**Rural residents**		**Civil servants**		**Workers**	
		aOR (95% CI)^*^	***P***	aOR (95% CI)^*^	***P***	aOR (95% CI)^*^	***P***
Pickles	Hardly	Reference		Reference		Reference	
	1–3 times/month	0.91 (0.47, 1.76)	0.782	1.07 (0.78, 1.48)	0.671	1.18 (0.82, 1.70)	0.385
	1–3 times/week	0.81 (0.38, 1.73)	0.585	1.30 (0.93, 1.82)	0.126	1.43 (0.98, 2.10)	0.064
	≥4 times/week	2.95 (1.36, 6.41)	0.006	0.78 (0.47, 1.30)	0.338	1.05 (0.60, 1.84)	0.879
Processed meats	Hardly	Reference		Reference		Reference	
	1–3 times/month	1.79 (0.73, 4.43)	0.205	1.33 (0.99, 1.77)	0.057	1.17 (0.85, 1.61)	0.348
	1–3 times/week	2.15 (0.78, 5.95)	0.139	1.31 (0.92, 1.86)	0.129	1.71 (1.06, 2.74)	0.027
	≥4 times/week	2.33 (0.44, 12.2)	0.318	1.01 (0.56, 1.83)	0.968	0.90 (0.32, 2.52)	0.837
Processed foods	Hardly	Reference		Reference		Reference	
	1–3 times/month	0.87 (0.27, 2.75)	0.807	1.44 (0.91, 2.28)	0.116	1.31 (0.86, 2.02)	0.212
	1–3 times/week	1.30 (0.48, 3.51)	0.601	1.42 (0.97, 2.07)	0.071	1.32 (0.90, 1.94)	0.154
	≥4 times/week	2.06 (0.70, 6.01)	0.188	1.31 (0.86, 1.99)	0.208	1.37 (0.84, 2.21)	0.202

* *Adjusted for age and sex, income, smoking, intake of red meat and poultry, BMI, and MAP*.

**Figure 3:**
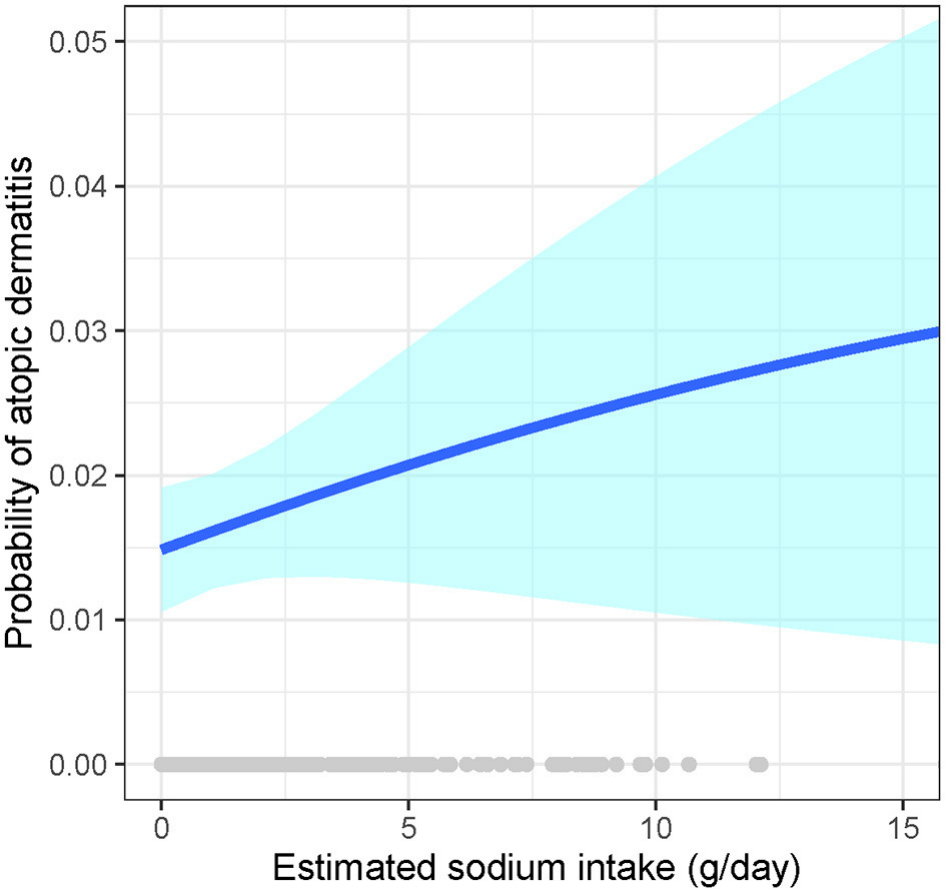
Association of the estimated sodium intake with atopic dermatitis. Generalized additive models with logit link function and binomial distribution to estimate the association of sodium intake and prevalence of AD, and cubic splines were used for smoothing. Adjustments included level-1 covariates (age, sex, income, smoking, intake of other foods, BMI, and MAP) and the random effects of level-2 (villages/institutions/factories) and level-3 (studies) units. The blue line signifies the probability of atopic dermatitis; the light-blue band signifies the 95% CI of the estimates.

We observed a dose-response association of the estimated intake of sodium chloride (NaCl)-based on the food contents with AD (Figure 3). We also determined the intake of sodium based on the spot urine using the Tanaka equations in 170 AD cases and 170 healthy controls matched by age and sex. However, the mean intake of sodium was 8.38 ± 1.59 g/d and 8.24 ± 1.65 g/d in AD patients and healthy controls, respectively, with no statistical significance (*P* = 0.627).

## Discussion

In this study, we identified processed food as a risk factor for AD in 15,062 Chinese adults based on a pooled analysis of three cross-sectional studies. Compared to non-consumers, the intakes of pickles and processed meats 1–3 times per week are associated with 35 and 44% additional risks of AD, respectively. However, the association of sodium intake with AD is not consistently based on different methods.

Processed foods (such as bacon, sausages, and pickles) generally have a more complex nutrition composition than natural foods, because almost all of them tend to be richer in sodium, nitrite, processed culinary ingredients, and microbial fermentation products. The complicated production processes and recipes for processed food vary substantially from different areas of the world (25–28). We hypothesized that one of the keys to explaining the association is salt (sodium) content. While the sodium contents vary across different kinds of processed food (for example, sodium in processed meat is 800–1000 mg/100 g worldwide on average, and some even exceed 1000 mg/100 g in some western and Asian countries), (29, 30) food processing is one of the main sources of sodium intake, accounting for 75% of the total sodium in the diet of American adults, as an example (31). Previous publications indicated that dietary salt restriction improved pediatric AD, and changing to low-salt table water also improved AD (32). Processed food as an important, excessive source of dietary sodium should not be ignored (33).

The pathogenesis of AD is attributed to immune-related abnormalities and skin barrier impairments (34). Our study identified sodium as a potential mechanism in that links processed food and AD. These results are consistent with findings from a previous *in-vitro* experiment showing that NaCl-mediated ionic signaling can induce the human alternative T cell to fate into the TH2 cell phenotype, (35) which is a characteristic of pathogenesis in AD (1). NaCl increases inflammatory response by enhancing TH2 signature cytokines interleukin-4 (IL-4) and IL-13 production (34) and it also has been confirmed to promote the differentiation of TH17 cells in polarizing cytokine conditions (36, 37). Long-term western diet pattern could aggravate skin inflammation diseases with TH 2 and TH17 pathway features in mice by dysregulating bile acid (38). Another study showed that administration of Lactobacillus murinus to mice could prevent the high expression of Th17 caused by high salt feeding (39). Given the important role of the mixed inflammatory reaction of TH 2 and TH17 in the Chinese population with AD, we speculate that a high-salt diet may have an association with the risk of AD by affecting immune-related pathways. Moreover, excessive sodium intake can also affect the epidermal barrier microenvironment. Sodium ions (Na+) can be combined with negatively charged glycosaminoglycans (GAGs) in the epidermis and distributed in the skin in an impermeable way of storing sodium (40, 41).

When intaking excessive sodium, the binding ability of GAGs and Na+ will be overloaded, thus resulting in a high osmotic pressure state and mast cell aggregation (42, 43). Furthermore, a recent *in-vivo* study has shown that knockdown of an epithelial sodium sensor, Nax, in mice resulted in the improvement of AD, (44) and the skin of patients with AD was found highly enriched in sodium content (35). Taken together, these findings reveal NaCl as an ionic checkpoint associated with both immune and skin barrier dysfunction in AD. In addition, since Staphylococcus aureus is known as tolerant of elevated NaCl conditions, (45) NaCl accumulation on the skin of patients could be another evidence for the skin microbial dysbiosis of AD.

Processed meat and red meat hold the main position in the rank of importance of food in their association with AD in our study. There was evidence that the intake of processed food could increase the abundance of sulfate-reducing bacteria (SRB) and upregulate the gene of sulfate reductase, resulting in more hydrogen sulfide (HS) production under SRB fermentation of sulfur-containing amino acids (46). HS could contribute to inflammation of the intestine and system, DNA damage, and genetic toxicity by changing the permeability of the intestinal mucosa at physiological concentrations (47). Undigested protein produces ammonia, branched-chain fatty acids, aromatic amino acids, and other harmful substances when digested in the large intestine (48). The above-mentioned metabolites are related to systemic inflammation, but further experimental exploration is needed to identify whether they are involved in the pathogenesis and development of AD.

The intake of red and processed meat is also related to the decline of bifidobacteria and short-chain fatty acids (SCFAs). SCFA can promote mucus secretion, protect and maintain the intestinal mucosal barrier and the intestinal microenvironment, and the decline of SCFA such as butyrate is closely related to the risk of many diseases (48–51). There are observational studies that show that AD patients are often accompanied by changes in the intestinal flora and its metabolites, including a decreased abundance of Bifidobacterium, Akkermania muciniphila, and Rumenococcus gnavus, and decreased fecal SCFAs and other metabolites (52).

The study has some strengths, including hypothesis-driven analysis, large sample size, comparability of methodologies across studies, and diagnosis of AD by dermatologists instead of self-reports. There are also limitations. First, the causal relationships cannot be established in cross-sectional studies. Second, the semi-quantitative method for exposure measurement was used, and the estimation of sodium intake was not accurate based on spot urine. FFQs could avoid problems related to changes in daily intake and cover a longer intake period, so they are mostly used in population-based research, but the accuracy of the semi-quantitative FFQ survey results and data was often influenced by individual factors (53, 54). To our knowledge, FFQ and spot were commonly used methods used in the estimation of sodium intake, while both of them had limitations in the estimation of 24 h sodium exposure according to previous studies. However, few studies compared them directly and simultaneously (49). As a pathway of easy application to estimate sodium intake, spot urine was often collected in clinical studies and different formulas were used to convert spot sodium to 24HUNa. Xu et al. found that Tanaka formula was relatively reliable for sodium estimation among the Chinese population in Shandong province, but this result should be explained with caution as there was great difference in salt intake and diet among Chinese people in different regions (55, 56). The newest study by Kelly et al. showed that all the spot urine methods were found to overestimate the actual content of sodium intake and had only a moderate correlation with 24HUNa, and it also found that FFQ presented poor accuracy in salt intake calculation. In other words, though the two methods were useful in some group levels, neither spot urine nor FFQ could be regarded as a unified pathway to provide accurate values in individuals (57). A more precise measurement of sodium intake through the collection of 24 h urine is warranted to confirm the association.

In conclusion, processed foods intake is associated with AD in Chinese adults, which might be related to sodium intake, but more accurate methods of dietary exposure assessment and FFQ were needed for further validation among Chinese people.

## Data Availability Statement

The raw data supporting the conclusions of this article will be made available by the authors, without undue reservation.

## Ethics Statement

The studies involving human participants were reviewed and approved by the Institutional Research Ethics Boards of Xiangya Hospital and Xiangya School of Public Health of Central South University (approval number# XYGW-2016-10) and Tongji Medical College of Huazhong University of Science and Technology has approved the studies (approval number# 2016-IEC-S128). The patients/participants provided their written informed consent to participate in this study.

## Author Contributions

YX, XC, and MS: conceptualization. YL and MS: formal analysis. YL, JS, DL, YD, ZH, MH, JT, SX, YX, XC, and MS: investigation and writing—review and editing. MS, DL, and MH: data curation. YL: writing—original draft preparation. YX, YL, and MS: revision. XC and SX: project administration. All authors read and approved the final manuscript.

## Funding

This work was supported by the Ministry of Science and Technology of People's Republic of China (2016YFC0900802).

## Conflict of Interest

The authors declare that the research was conducted in the absence of any commercial or financial relationships that could be construed as a potential conflict of interest.

## Publisher's Note

All claims expressed in this article are solely those of the authors and do not necessarily represent those of their affiliated organizations, or those of the publisher, the editors and the reviewers. Any product that may be evaluated in this article, or claim that may be made by its manufacturer, is not guaranteed or endorsed by the publisher.
